# HPV-16 virions can remain infectious for 2 weeks on senescent cells but require cell cycle re-activation to allow virus entry

**DOI:** 10.1038/s41598-017-18809-6

**Published:** 2018-01-16

**Authors:** Justyna Broniarczyk, Nadja Ring, Paola Massimi, Mauro Giacca, Lawrence Banks

**Affiliations:** 1 International Centre for Genetic Engineering and Biotechnology, Padriciano 99, I-34149 Trieste, Italy; 2 Department of Molecular Virology, Adam Mickiewicz University, Umultowska 89, 61-614 Poznan, Poland

## Abstract

Successful infection with Human Papillomaviruses requires mitosis, when incoming viral genomes gain access to nuclear components. However, very little is known about how long HPV particles can remain infectious in non-dividing cells or in which cellular compartments these viruses may reside. To investigate these questions we have used BJ cells as a reversible model of senescence and show that HPV-16 can only infect early-passage proliferating cells. Late-passage senescent cells are resistant to HPV infection, but this can be reversed by inducing cell cycle re-entry with a p53 siRNA. In senescent cells we find that efficient virus entry can be attained upon cell cycle re-entry 16 days after infection, demonstrating that HPV can persist for 2 weeks prior to induction of mitosis. However, exposing cells to anti-HPV-16 L1 neutralising antibody blocks infection at these late time points, suggesting that the virions reside near the cell surface. Indeed, immunofluorescence analysis shows that virions accumulate on the cell surface of senescent cells and only enter endocytic vesicles upon stimulation with p53 siRNA. These results demonstrate that HPV-16 virions can remain viable on a non-dividing cell for extended periods of time, but are nonetheless vulnerable to antibody-induced neutralisation throughout.

## Introduction

Human Papillomaviruses (HPVs) are major human pathogens and the causative agents of a number of important human malignancies, with cervical cancer being the most important^1,2^. The viruses replicate in differentiating epithelia, where the virus initially gains access to the basal cell compartment, which is thought to occur through microtraumas in the skin. Once infected, the basal keratinocyte begins to differentiate and the combined action of the viral oncoproteins, E6 and E7, promotes cell cycle entry and replication of the viral genomes. Ultimately this process results in the production of new infectious virus particles in the upper terminally-differentiated layers of the skin^3,4^. In rare cases this infectious cycle is perturbed, and over a number of years malignancies can subsequently arise.

The viral capsid contains the double stranded viral genome of approximately 8 kb, which is enclosed by the viral coat proteins L1 and L2^5,6^. Whilst both proteins play essential functions in capsid assembly and virus entry, the viral L2 protein appears to be the most important for ensuring delivery of the viral genome to the infected cell nucleus, where viral gene expression can initiate^7,8^. The whole process of virus infection involves multiple steps. After the initial attachment of incoming virions to the extracellular matrix^9,10^, there is a structural alteration to the viral capsid, which allows binding to the target cell and subsequent endocytic uptake^11,12^. During the process of endocytic maturation and acidification the capsid begins to disassemble, and at some point in this process the L2 proteins become partially exposed to the cytoplasmic side of the endocytic vesicle^13,14^. This exposure of L2 plays a critical role in recruiting different components of the endocytic cargo sorting machinery, which includes components of the retromer complex^15,16^ and members of the sorting nexin protein family^17,18^. Components of the ESCRT machinery also seem to play an important role in these early steps of infectious entry^19–21^ and eventually, through the action of cyclophilins, the L1 protein becomes largely dissociated from the L2/DNA complex and is processed to the lysosomal compartments and degraded^22^, whilst the L2/DNA complex is trafficked to the trans-Golgi network^23^. Only upon the initiation of mitosis and nuclear envelope breakdown does the L2/DNA complex, accompanied by a small amount of residual L1, then gain access to the nucleus where the viral genome ultimately resides at PML oncogenic domains (PODs)^24–26^. Viral gene expression is believed to initiate at these domains and the onset of a new round of viral genome amplification and viral production proceeds^7,8^.

This whole entry process is thought to be rather slow, often taking many hours, although if infection occurs at a point when the cells are about to divide then entry into the nucleus can be much faster^27^. Virus uptake itself is also believed to be dependent upon growth factor signalling, and there is some evidence to suggest that virus entry may in part be linked to growth factor receptor internalisation, suggesting that there is preferential entry of the virus into proliferating cells^28,29^. Nonetheless a critical step in this whole pathway is the initiation of mitosis, without which the virus cannot gain access to the nucleus and the infection fails^24,25^.

Whilst HPV virions are known to be quite resilient, there is very little information on how long they can remain infectious once exposed to their target cells, or for how long, or where, such viruses might reside in a cell that is not undergoing mitosis. In order to begin to provide some answers to these questions we have made use of a model of reversible replicative cellular senescence. BJ cells are fibroblasts that undergo replicative senescence following extended periods of passage in tissue culture, a process first described in 1965^30^. However these cells can be stimulated to re-enter the cell cycle and divide further by transfecting them with siRNAs that knock down components of the p53 signalling pathway, including p53 itself and p21^31^. Using this model system we have investigated how long virus particles can remain viable once exposed to senescent cells before they are triggered to re-enter the cell cycle. We have also ascertained where virions can reside long-term in the cell when the cell is outside the cell cycle and before induction of mitosis. To our surprise, we find that under these conditions virions can remain infectious for several weeks, not in internal vesicular compartments but instead retained on the cell surface in a location accessible to neutralising antibodies.

## Materials and Methods

### Cell lines and transfections

Human diploid foreskin fibroblasts (BJ, ATCC CRL-2522) were a generous gift from Fabrizio D’Adda Di Fagagna’s laboratory and were cultured in MEM with Glutamax supplemented with 10% fetal bovine serum, penicillin-streptomycin (1%), glutamine (2 mM), non-essential amino acids (10 mM) and sodium pyruvate (1 mM). In order to be able to observe viral persistence in non-dividing cells, BJ fibroblasts were serially cultured at 37 °C and 95% humidity with 5% CO_2_ to replicative senescence. At this point, less than 10% of the cells incorporated EdU over the course of a 24 h period, with the majority of cells displaying a cell cycle arrest. Cells were either subcultured (using 0.05% trypsin-EDTA) or submitted to a medium change approximately every 4 days and kept at a maximum confluence of approximately 90%. HeLa cells were maintained in DMEM supplemented with 10% fetal bovine serum, penicillin-streptomycin (1%), glutamine (2 mM).

BJ cells were transfected following a reverse transfection protocol using Lipofectamine RNAiMAX (Invitrogen) with siRNAs against p53 (Sigma) or non-targeting siRNA (control siRNA, Dharmacon) as a control. In order to monitor transfection efficiencies, an siRNA targeting the ubiquitin complex (siUBC, Dharmacon) was used in all assays, which is cytotoxic after successful transfection, and verified transfection efficiencies of over 90%.

### Cell cycle analysis with flow cytometry

4 × 10^5^ senescent or replicating BJ fibroblasts were plated on 6 cm dishes. Senescent cells were reverse transfected with control or p53 siRNA to a final concentration of 50 nM. Cells were harvested 72 h post-transfection and cell cycle analysis was performed with a fluorescence-activated cell sorter (FACS) by measuring DNA content using propidium iodide staining as described previously^32^.

### EdU incorporation assay

1.5 × 10^3^ senescent BJ cells were reverse transfected in a 384-well plate with control siRNA, p53 siRNA and UBC siRNA as a control for cell viability. After 48 h 10 µM EdU was added to the cells and after 24 h the cells were fixed and stained with Click-IT (Thermo Fisher Scientific) following the manufacturer’s protocol.

### Western blot

1.3 × 10^5^ senescent BJ cells were untreated or reverse transfected in a 6-well plate with control siRNA or p53 siRNA and harvested in RIPA buffer after 72 h. Equal amounts of protein were separated using SDS-PAGE and transferred to a nitrocellulose membrane. The p53 protein was detected using the anti-p53 (DO-1) mouse monoclonal antibody (sc-126, Santa Cruz Biotechnology), and the loading control alpha-actinin was detected using the rabbit anti-alpha-actinin (H-300) polyclonal antibody (sc-15335, Santa Cruz Biotechnology). Western blots were developed using appropriate secondary antibodies conjugated to HRP followed by ECL detection.

### PsVs production

Different HPV-PsVs containing a luciferase reporter plasmid were generated in 293TT cells as described previously^33^. Purity and capsid protein content were determined by SDS-PAGE and Coomassie Brilliant blue staining, while the encapsidated DNA was quantified using Real-Time PCR and the copy number was calculated using a standard curve of reporter plasmid DNA. AF488-labelled PsVs were prepared using the Alexa Fluor 488 Protein labelling kit (Molecular Probes).

### Infectivity assay

BJ senescent cells were seeded in 96-well plates at a density of 4 × 10^3^ cells per well, and exposed to 100 vge/cell of luciferase reporter positive PsVs. 1, 13 or 27 days post-infection (with cells subjected to several medium changes), cell division was activated by transfection with p53 siRNA and harvested 72 h later (constituting 4, 16 or 30 days post-infection). Infection was monitored by luminometric analysis of firefly luciferase activity using the Luciferase Assay System (Promega). For each assay, PsVs infection in the presence of control siRNA was normalized to 100%, which was then used to calculate the percentage increase following transfection of the cells with the p53 siRNA.

To compare HPV-16 PsVs infectivity in different cell lines, BJ early passage cells and HeLa cells were seeded in a 96-well plate at a density of 2.5 × 10^3^ cells per well, and exposed to 100 vge/cell of luciferase reporter-positive PsVs. Two days post-infection cells were lysed and analysed for luminescence as an indication of efficient infection. Throughout, equal amounts of total cell protein extract were used in the luciferase measurements.

### Transferrin uptake

For analysis of transferrin internalisation, senescent cells were reverse transfected with control siRNA and p53 siRNA and plated on 0.01% poly-L-lysine-coated LabTek 8-well chamber slides (Thermo Fisher Scientific) at a density of 1.4 × 10^4^ cells per well. 72 h after transfection cells were incubated for 30 min at 37 °C in serum-free MEM Glutamax with addition of 50 µg/ml AF647 labelled transferrin (Molecular Probes). Following incubation, cells were washed several times with PBS and fixed in 3.7% paraformaldehyde (PFA) for 15 min for fluorescence microscopy analysis. Cells were incubated for 1 h at room temperature with 10% goat serum and later EEA1 detection was performed using rabbit anti-EEA1 (C45B10, Cell Signaling Technology) and secondary goat anti-rabbit AF546-conjugated antibody (Molecular Probes). Slides were visualised using a Zeiss Axiovert 100 M microscope attached to an LSM 510 confocal unit. Transferrin uptake was analysed using ImageJ. 15 fields acquired with a confocal microscope were analysed per condition, with over 130 cells in total analysed for both control and treated samples. The mean intensity of the transferrin staining was measured for each individual cell, disregarding the cell area. A second analysis was performed whereby the integrated intensity (the product of mean intensity and cell area) was used to represent the total transferrin content of each cell.

### Immunofluorescence

BJ senescent cells were reverse transfected with control siRNA and p53 siRNA and seeded on LabTek 8-well chambers slides (Thermo Fisher Scientific) at a density of 1.4 × 10^4^ cells per well. One day after, the cells were exposed to 500 vge/cell of AF488-labelled PsVs. 48 h after infection and 72 h after transfection, cells were fixed in 3.7% paraformaldehyde for 15 min, incubated for 1 h with 10% goat serum and then stained using antibodies for early endosomes (rabbit anti-EEA1, C45B10, Cell Signaling Technology) and lysosomal markers (mouse anti-LAMP1, D4O1S, Cell Signaling Technology). Donkey anti-rabbit 594 and goat anti-mouse rhodamine were used as secondary antibodies (Molecular Probes). Slides were visualised using a Zeiss Axiovert 100 M microscope attached to an LSM 510 confocal unit.

### Neutralisation assay

Neutralisation assays were performed using the anti-L1 neutralising antibody (H16.V5), which was kindly provided by Neil Christensen^34^. In infectivity assays neutralising antibody was added to the medium (dilution of 1:1000) 24 h prior to adding siRNA, and infectivity was measured at 16 days post-infection as described above. In immunofluorescence assays cells were transfected and 1 day later cells were infected, and 2 days post-transfection the anti-L1 neutralising antibody was added to the medium at a dilution of 1:200. Cells were fixed 3 days post-transfection and neutralising antibody was visualised using secondary goat anti-mouse rhodamine antibody (Molecular Probes).

### Statistics

All experiments were performed at least in triplicate, and data are shown as mean and standard deviation. Statistical significance was calculated using the GraphPad prism 6 software. To compare two groups the Student’s t-test was performed, to compare several samples a 1-way ANOVA calculation was followed by Dunnett’s multiple comparison test. A p-value below 0.05 was considered statistically significant and throughout the p-values have been defined as follows *p < 0.05, **p < 0.01, ***p < 0.001, ****p < 0.0001, while “ns” represents a non-significant p-value above 0.05.

## Results

**Figure 1:**
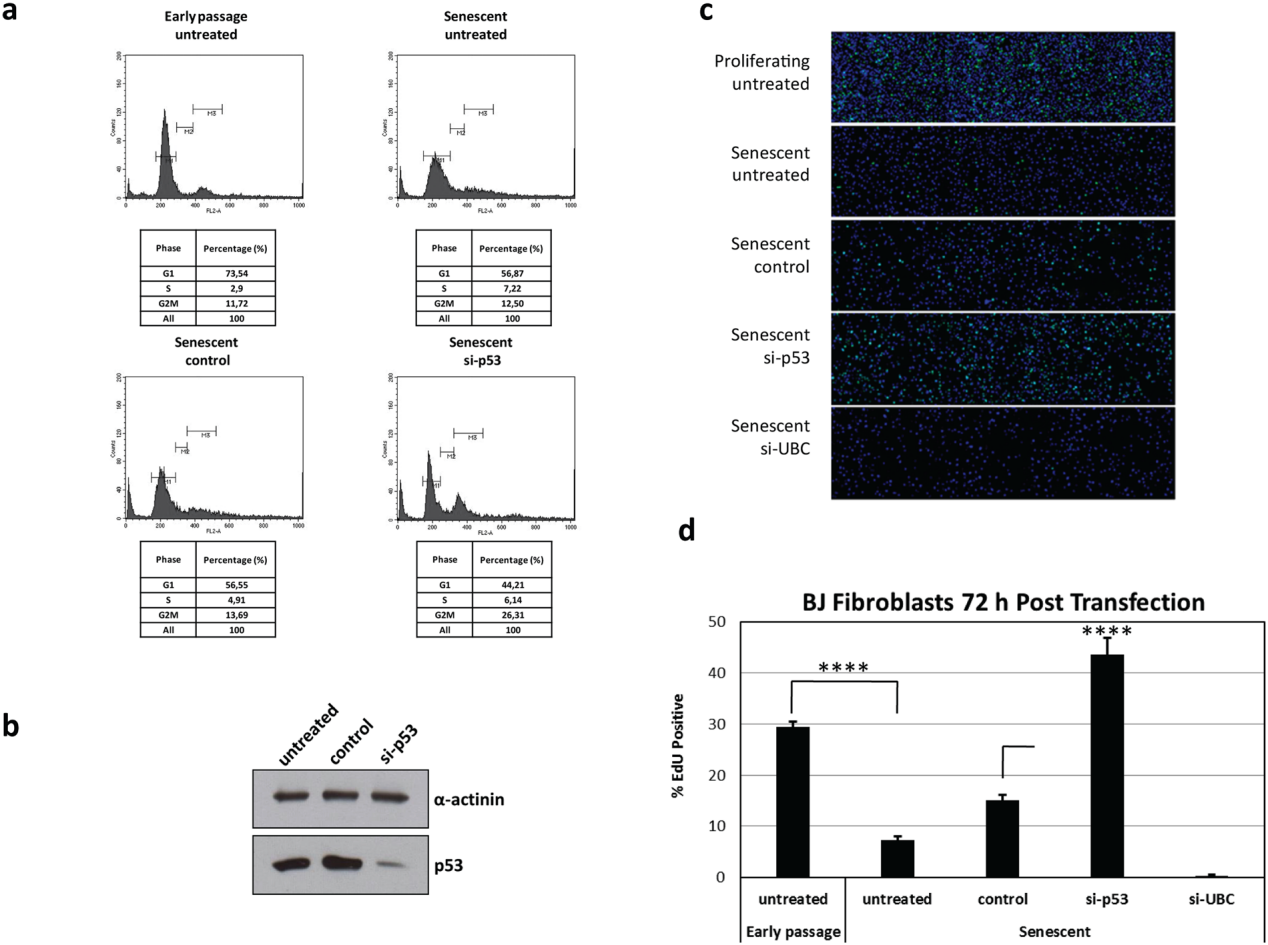
p53 siRNA induces senescent BJ cells to re-enter the cell cycle and re-commence proliferation. (**a**) Flow cytometry analysis of BJ fibroblast cell cycle. Early passage untreated BJ fibroblasts and late passage (senescent) BJ fibroblasts (untreated, or transfected with control siRNA or p53 siRNA) were harvested after 72 h and the cell cycle was analysed using propidium iodide incorporation. Note that senescent cells are arrested in G0 and appear to re-enter the cell cycle following transfection with si-p53. (**b**) Western blot showing p53 expression in senescent BJ cells. Late passage (senescent) BJ fibroblasts (untreated, or transfected with control siRNA or p53 siRNA) were harvested after 72 h and protein analysis was performed. Alpha-actinin was used as a loading control. Note that the levels of p53 in senescent fibroblasts are efficiently reduced by the siRNA transfection. (**c**) Cell proliferation analysis using an EdU incorporation assay. Early and late passage (senescent) fibroblasts were plated (untreated or reverse transfected with control siRNA, p53 siRNA or siRNA targeting Ubiquitin C (si-UBC)). EdU was added to cells 48 h after transfection, cells were labeled for 24 h and then fixed. The incorporated EdU was stained with Click-IT (green), with cell nuclei counter-stained with Hoechst 33342 (blue). (**d**) Quantification of EdU incorporation. Plates were acquired using the Image Xpress micro microscope (Molecular Devices) and analysed using MetaXpress version 5.3.0.5 to determine percentage of EdU incorporation. The results are expressed as the mean of three independent experiments, and the standard deviations are shown. Note that cells treated with p53 siRNA (si-p53) have a higher proliferative ability than cells transfected with control siRNA.

Previous studies have shown that completion of mitosis is an absolute requirement for allowing completion of HPV-16 infectious entry^24,25^. However we have been interested in understanding where incoming virions reside until the onset of mitosis and for how long such virions can remain viable. In order to address these questions we made use of a well-established model of cellular senescence. In the BJ cell model, early passage cells grow rapidly, but following extensive periods of passage they enter senescence. This is indeed confirmed in Fig. 1a, where it can be seen that late passage cells show very low levels of DNA replication. However, transfection of a p53 siRNA, the efficacy of which was verified by Western blotting in Fig. 1b, efficiently reverses the senescent phenotype, and as can be seen from the EdU incorporation assay in Fig. 1c and d these cells re-enter the cell cycle and activate DNA replication.

**Figure 2:**
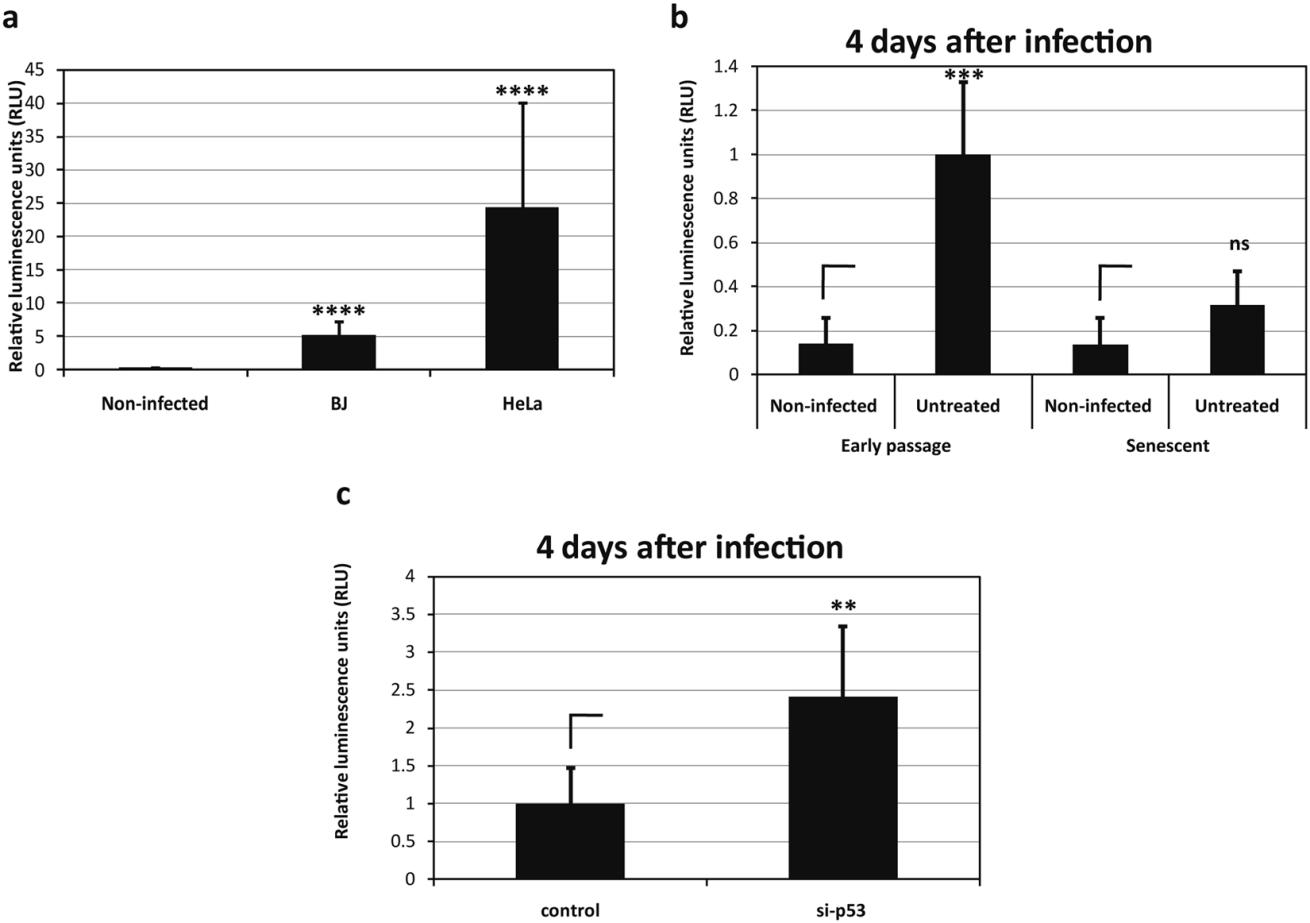
HPV-16 PsV infection of senescent cells is restored following transfection of p53 siRNA. (**a**) Infectivity assay in early passage BJ and HeLa cells. Early passage untreated BJ fibroblasts and Hela cells were infected with HPV-16 PsVs. Two days post-infection cells were lysed and analysed for luminescence as an indication of efficient infection. The results are expressed as the means from at least three independent experiments, and the standard deviations are shown. (**b**) Infectivity assay in untreated early and late passage BJ cells. Early passage and late passage (senescent) untreated BJ cells were infected with HPV-16 PsVs carrying a luciferase reporter plasmid. Four days post infection cells were lysed and analysed for luminescence as an indication of efficient infection. The results are expressed as the means from at least three independent experiments, and the standard deviations are shown. Note that only early passage untreated BJ cells are efficiently transduced using the HPV-16 PsVs, while untreated late passage (senescent) cells are not. (**c**) Infectivity assay in transfected late passage BJ cells. Senescent BJ fibroblasts were infected with HPV-16 PsVs carrying a luciferase reporter plasmid and 24 h later transfected with control siRNA, or p53 siRNA. Four days post-infection cells were lysed and analysed for luminescence as an indication of efficient infection. The results are expressed as the mean of at least three independent experiments, and the standard deviations are shown. Note that late passage (senescent) cells are only efficiently transduced if stimulated to re-enter the cell cycle through p53 siRNA transfection.

Using this model system, we first compared the ability of HPV-16 PsVs carrying a luciferase reporter construct to infect early passage BJ fibroblasts cells and cervical tumour-derived HeLa cells. The results in Fig. 2a show that the BJ fibroblasts are susceptible to infection with the HPV-16 PsVs, although somewhat less so than HeLa cells. Having found that BJ fibroblasts can be used in such infection assays, we next compared the ability of HPV-16 PsVs carrying a luciferase reporter gene to infect early and late passage BJ cells. As can be seen from Fig. 2b, early passage BJ cells are efficiently transduced using the HPV-16 PsVs but the late passage senescent cells are not, which is consistent with their not undergoing cell division. These assays were then repeated in the presence of p53 siRNA, and as can be seen from Fig. 2c, this renders the late passage cells susceptible to HPV-16 infection and promotes transduction of the reporter construct, consistent with reactivation of cell division, allowing the incoming viruses to gain access to the nucleus during mitosis.

**Figure 3:**
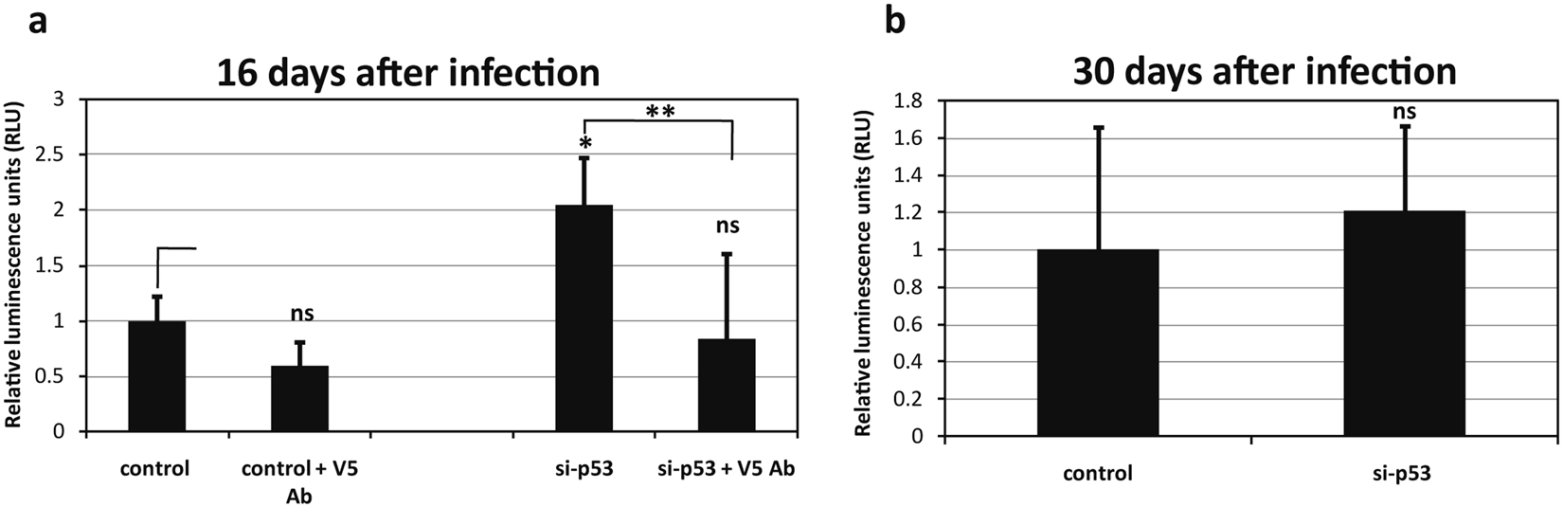
HPV-16 PsVs are susceptible to antibody neutralization for 2 weeks on senescent cells. (**a**) Infectivity assay at 16 days post-infection. Senescent BJ cells were infected with HPV-16 PsVs containing the luciferase reporter gene. After 13 days, cell division was activated by transfection with p53 siRNA. Cells were harvested 72 h after transfection and luciferase activity was measured to assess the levels of infection. Additionally, the day before adding the siRNA the cells were incubated in the presence or absence of the H16.V5 neutralising antibody (1:200). The results are expressed as the means from at least three independent experiments, and the standard deviations are shown. (**b**) Infectivity assay at 30 days post-infection. Senescent BJ cells were infected with HPV-16 PsVs containing the luciferase reporter gene. After 27 days cell division was activated by transfection with p53 siRNA. Cells were harvested 72 h after transfection and luciferase activity was measured to assess the levels of infection. Note that the virus can survive on the cell for 2 weeks, but not 30 days, waiting for the initiation of mitosis to allow its entry into the nucleus. The neutralising antibody can block this infection, showing that the virus is localised in a position that is accessible to the neutralising antibody.

We were next interested in investigating how long the virus can survive post-infection before transfection of the p53 siRNA. To do this, late passage BJ cells were exposed to HPV-16 PsVs, and after 24 h the cells were washed and medium replaced. The cells were then cultured for 13 days and 27 days, after which they were transfected with the p53 siRNA to promote cell cycle re-entry. HPV-16 infectious entry was then measured 48 h later by extracting the cells and measuring luciferase activity. It can be seen from Fig. 3a that transfection of p53 siRNA into senescent cells can stimulate transduction of HPV-16 PsVs 16 days after exposure to the virus. However, if the cells are exposed 27 days prior to transfection of the p53 siRNA, then no PsV transduction occurs (Fig. 3b). This indicates that wherever the incoming HPV-16 PsVs reside for these extended periods of time in the senescent cells, they can remain viable and capable of transducing the reporter construct upon stimulation of cell cycle re-entry for at least 2 weeks, but most likely for no longer than 4 weeks. This suggests that in a normal virus infection cells need not be immediately proliferating to still be susceptible to virus infection, and that quite extended periods of time might pass before proliferation and mitosis commences.

**Figure 4:**
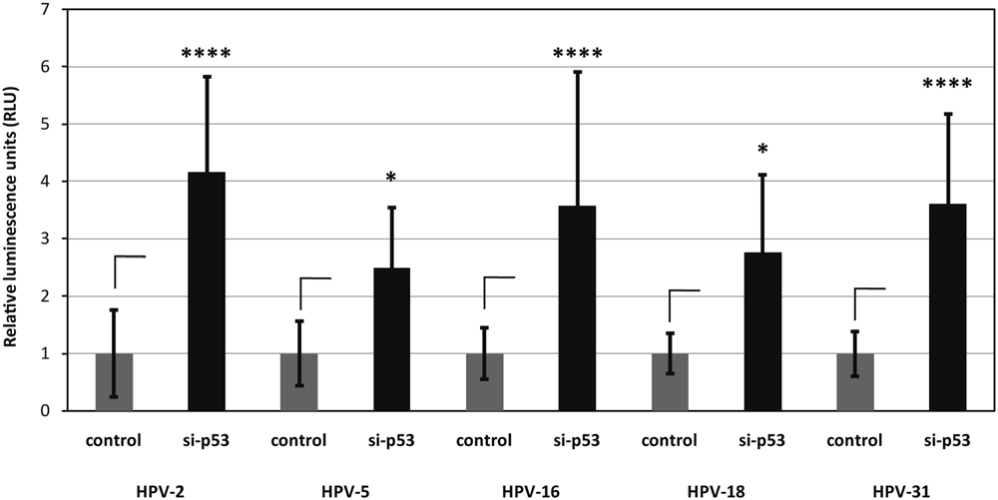
Comparison of the transduction efficiencies of different HPV types in siRNA p53 transfected senescent BJ fibroblasts. Senescent BJ fibroblasts were infected with HPV-2, HPV-5, HPV-16, HPV-18 and HPV-31 PsVs carrying a luciferase reporter plasmid and 24 h later transfected with control siRNA, or p53 siRNA. Four days post-infection the cells were lysed and luciferase activity measured. The results are expressed as the mean of at least three independent experiments, and the standard deviations are shown.

In order to determine where HPV-16 PsVs might reside for such extended periods of time we first analysed whether the virus was susceptible to antibody-induced neutralisation. To do this, late passage cells were exposed to PsVs as before, and left for a total of 16 days. The anti-L1 neutralising antibody (H16.V5) was added to the cells at 12 days post-infection, and at 13 days post-infection cells were transfected with the p53 siRNA. As can also be seen from Fig. 3, HPV-16 PsVs are readily neutralised by addition of the anti-L1 antibody, even after exposing the cells to virus 12 days earlier. These results demonstrate that the virus is most likely residing on, or very close to the cell surface, in a compartment that can be accessed by the anti-L1 antibody. We were then interested in investigating whether a panel of other HPV types could similarly infect senescent cells when they are stimulated back into the cell cycle following transfection of a p53 siRNA. To do this, late passage BJ cells were infected with HPV-2, HPV-5, HPV-16, HPV-18 and HPV-31 PsVs in the presence or absence of p53 siRNA. After 72 h the cells were harvested and luciferase activity measured. The results in Fig. 4 demonstrate a similar stimulation of luciferase transduction with all HPV types tested following transfection of p53 siRNA, indicating similar requirements for infectious entry amongst these different HPV types.

**Figure 5:**
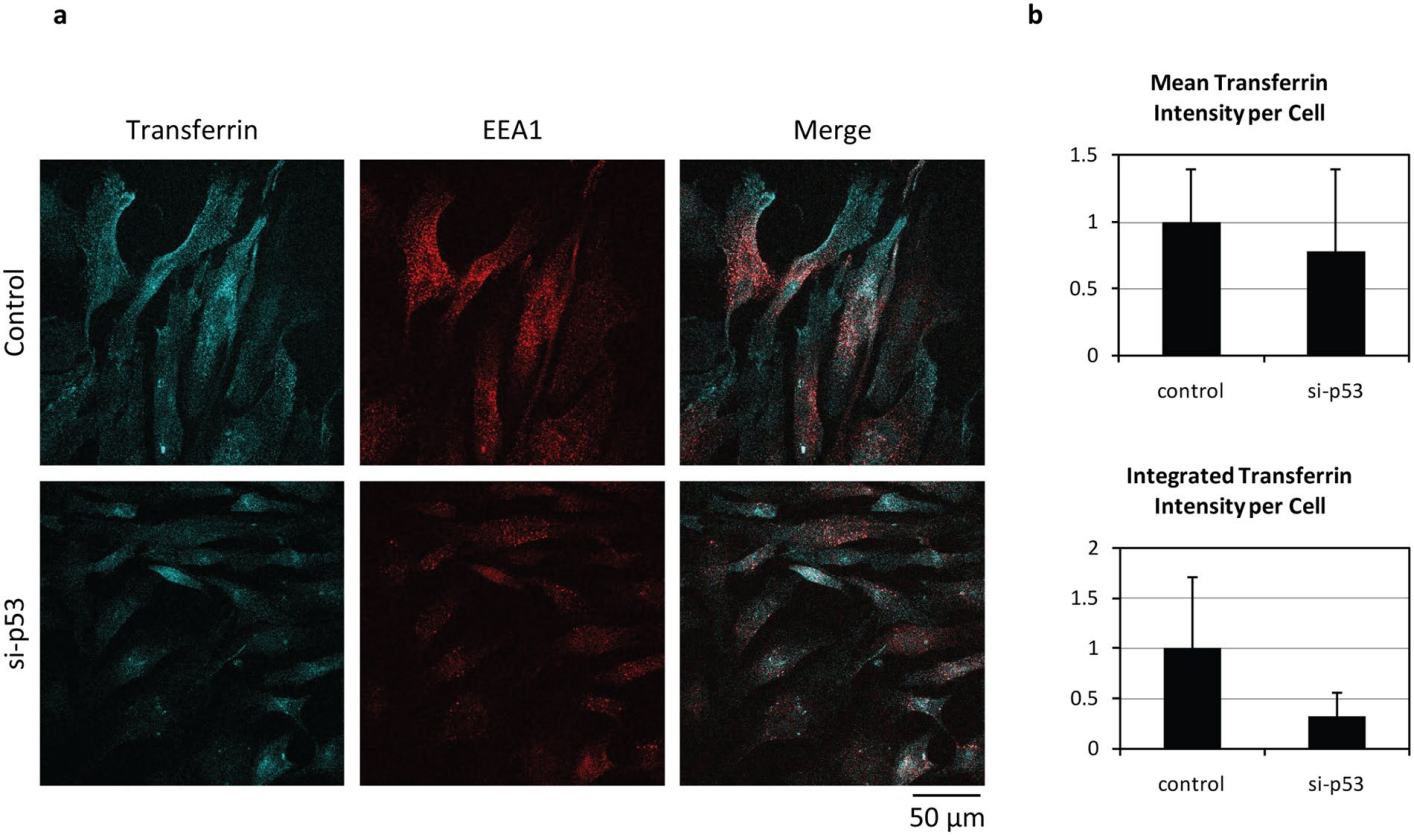
Transferrin uptake in senescent cells is not improved by re-stimulating cell cycle entry. Senescent BJ cells were transfected with control siRNA and p53 siRNA. 72 h post-transfection cells were incubated with AF647-labelled transferrin for 30 minutes before cell fixation. Transferrin (blue) co-localises with early endosomes EEA1 (red) 30 minutes after its uptake. Representative images are shown in panel (**a**) and quantification of transferrin uptake is shown in panel (**b**), calculated as average intensity and integrated intensity (representing total amount). Note that transferrin uptake and distribution is similar in senescent (control siRNA) and proliferating cells (si-p53), with no statistically significant differences in uptake regardless of analysis method.

Obviously an important concern from these analyses was whether or not the senescent cells were still undergoing endocytosis. In order to monitor this we analysed transferrin uptake in senescent cells and in cells transfected with p53 siRNA. At 72 h post-transfection cells were incubated with AF647-labelled transferrin for 30 minutes before cell fixation, and then stained for the early endosomal marker EEA1. As can be seen from Fig. 5, there is no major change in endocytic uptake of transferrin in the p53 siRNA-transfected senescent cells, indicating that lack of virus uptake is not due to the simple shutting down of endocytic transport pathways. This is supported by the quantifications from multiple assays when looking at the average transferrin intensity per cell, but also when integrating transferrin intensity, where the average intensity of transferrin is multiplied by the cell area.

**Figure 6:**
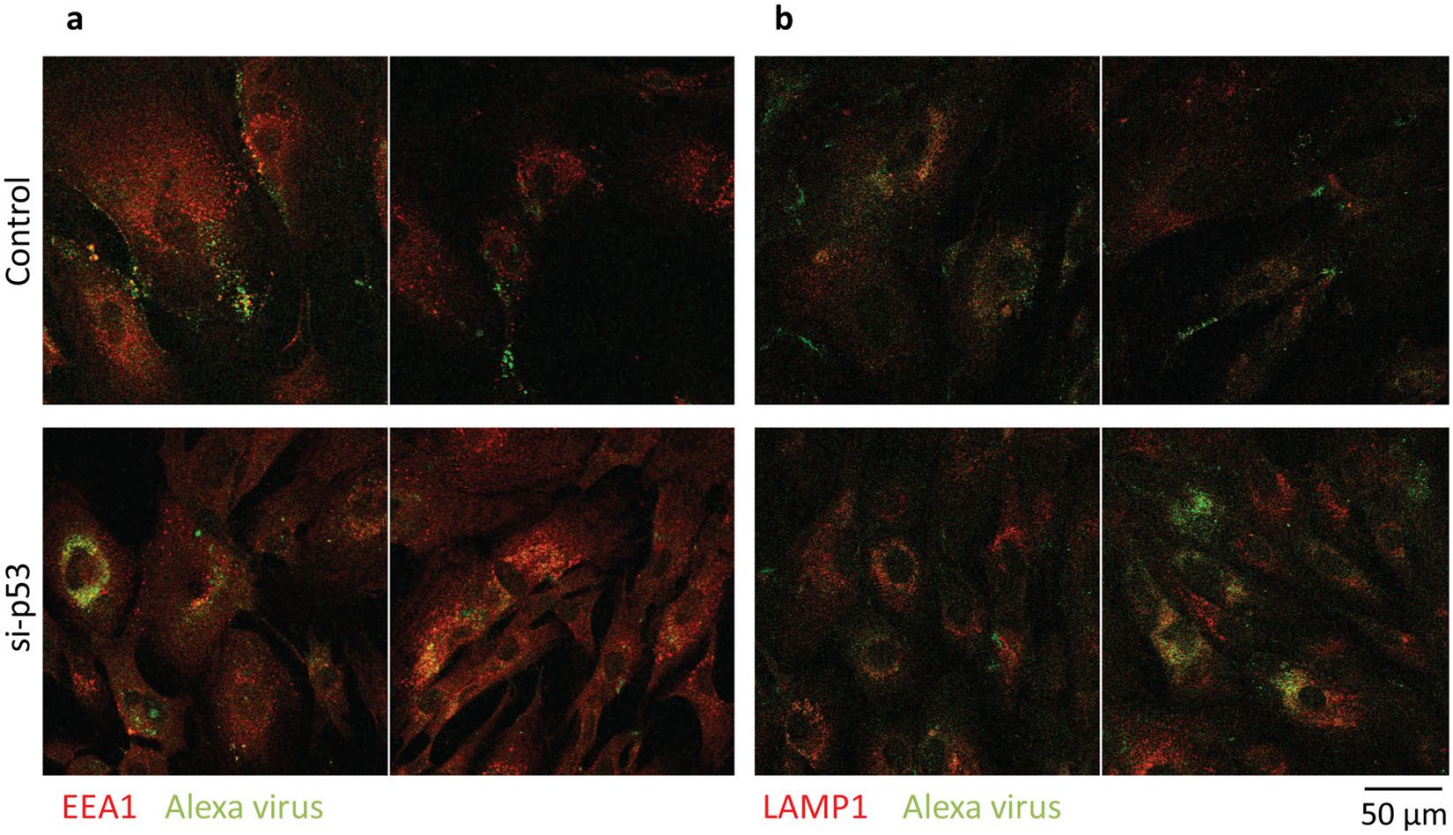
HPV-16 PsVs trafficking to early and late endosomes is affected in senescent cells. Senescent BJ cells were transfected with control siRNA and p53 siRNA. One day after transfection, cells were exposed to Alexa Fluor 488-labelled HPV-16 PsVs (green) for 48 h then fixed and stained for endogenous EEA-1 using rabbit antibody against EEA-1 and detected using Alexa Fluor 594-conjugated donkey anti-rabbit antibody (red) (**a**) and for endogenous LAMP1 using mouse antibody against LAMP1 and detected using rhodamine red-conjugated goat anti-mouse antibody (red) (**b**). Shown are two representative merged images for each condition. Note that in senescent cells (control) the virus is mainly localised on the surface of the cells and shows very little co-localisation with early endosomes (**a**) and late endosomes (**b**) in comparison with proliferating cells (si-p53).

Having found that the HPV-16 PsVs remained antibody-neutralisable for an extended period post-infection in the senescent cells, we then proceeded to investigate in more detail where such virions might reside prior to cell cycle re-entry. We first performed a series of analyses using Alexa Fluor-labelled (AF488) HPV-16 PsVs, where cells were first reverse transfected with the siRNAs, infected after 24 h and fixed 48 h post-infection. Cells were then stained for the endosomal marker EEA1 and the lysosomal marker LAMP1. The results in Fig. 6 demonstrate no extensive co-localisation between the AF488-labelled virus and either EEA1 or LAMP1 in the senescent cells, consistent with the virus not entering these cells. Indeed, in many cases the virus appears to accumulate at the cell periphery. In contrast, in cells transfected with the p53 siRNA there is clear evidence of virus co-localisation with EEA1 and LAMP1, demonstrating that stimulation of cell cycle re-entry also stimulates endocytic uptake of the HPV-16 PsVs.

**Figure 7:**
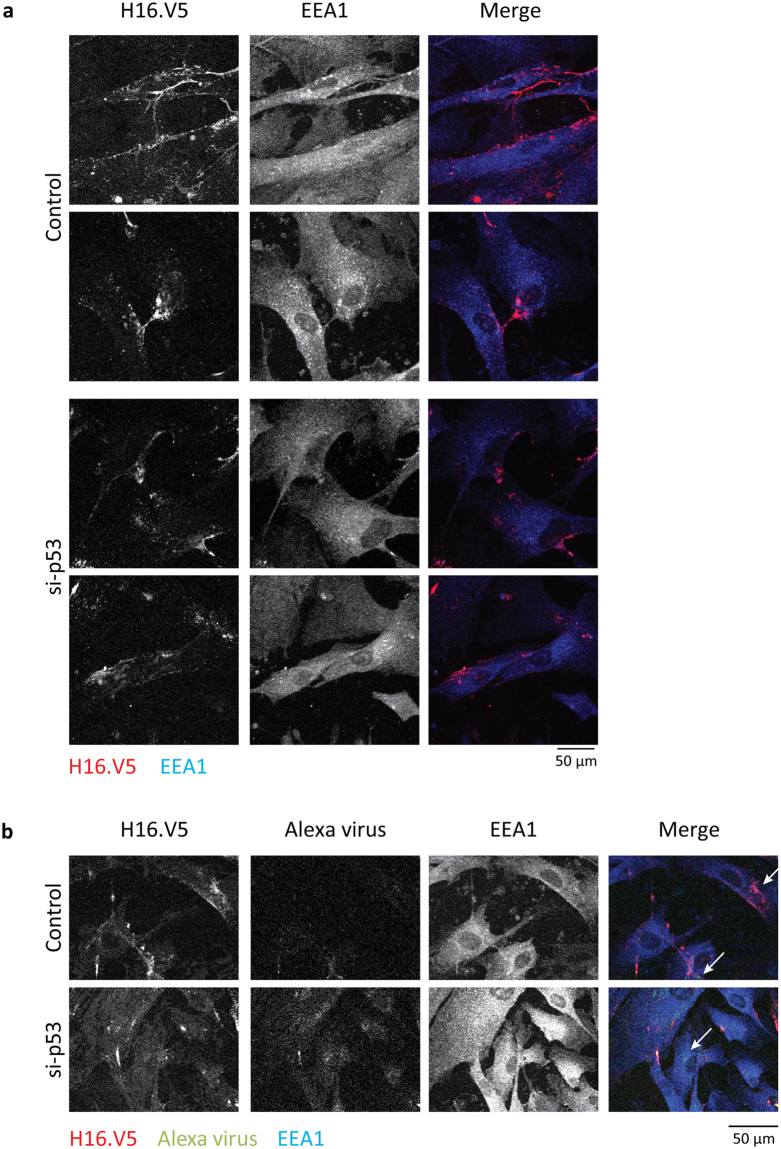
HPV-16 PsVs in senescent cells are accessible to neutralising antibody. (**a**) Anti-L1 (H16.V5) neutralising antibody binds to the surface of senescent and proliferating cells. Senescent BJ cells were transfected with control siRNA and p53 siRNA. 48 h after transfection cells were incubated with anti-L1 neutralising antibody for 24 h. Then cells were fixed and neutralising antibody was detected with goat anti-mouse rhodamine antibody (red) and rabbit antibody against EEA-1, and detected using Alexa Fluor 647-conjugated donkey anti-rabbit (blue). Two examples are shown for each condition. (**b**) In infected senescent cells the H16.V5 neutralising antibody co-localises with the virus on the surface of cells. One day after siRNA transfection, AF488-labelled HPV-16 PsVs (green) were added to the cells. 24 h after infection cells were incubated overnight with neutralising H16.V5 antibody, fixed and stained as above. Note that in senescent cells transfected with the control siRNA, H16.V5 neutralising antibody co-localises with the virus on the cell surface (indicated by arrows). In cells that were transfected with the p53 siRNA there is very little co-localisation between the virus and neutralising antibody and virus is visible within the cells, as indicated by the arrows, suggesting that virus entry is stimulated post–transfection of the p53 siRNA.

The above results indicate that HPV-16 PsVs most probably reside on the surface of the senescent cells, and are only endocytosed once the cells are stimulated to re-enter the cell cycle following the transfection of the p53 siRNA. We then wanted to investigate whether we could visualise the anti-L1 neutralising antibody (H16.V5) co-localising with the surface-bound HPV-16 PsVs in the senescent cells. To do this, the cells were transfected with control or p53 siRNA and after 24 h infected with AF488-labelled virus; after an additional 24 h the medium was changed and non-fixed, non-permeabilised cells were incubated with the anti-L1 antibody. Finally, one day later, the cells were washed and fixed and the anti-L1 neutralising antibody was detected by staining with rhodamine-conjugated goat anti-mouse antibody. As can be seen from Fig. 7a, the anti-L1 neutralising antibody binds to the surface of uninfected BJ cells, and this pattern does not change significantly following transfection of the p53 siRNA. This indicates that these antibodies can bind to the surface of these senescent cells probably through Fc receptors on the cell surface. However, in cells that have been infected with the HPV-16 PsVs and transfected with the control siRNA (Fig. 7b) it is quite clear that the AF488-labelled HPV-16 PsVs are located mostly on the cell surface, and there is clear co-localisation in many cases with the anti-L1 neutralising antibody. These results thus confirm that HPV-16 PsVs in the non-dividing cells are readily accessible to the neutralising antibody owing to the virus accumulating at a surface-exposed location. In contrast, in cells transfected with the p53 siRNA, the bulk of the HPV-16 PsVs have entered the cells while the anti-L1 antibody is still present on the cell surface. Under these conditions there is very little co-localisation between the AF488-labelled HPV-16 PsVs and the anti-L1 antibody. These results demonstrate that in non-dividing senescent cells HPV-16 PsVs remain bound to the cell surface, in a location accessible to neutralising antibodies. However, following stimulation of cell cycle re-entry there is rapid internalisation of the HPV-16 PsVs which are then no longer accessible to the anti-L1 antibody.

## Discussion

HPV infectious entry involves a complex series of steps, which includes interaction with extracellular matrix, structural remodelling of the virion, and receptor attachment, followed by endocytosis and the recruitment of different components of the cellular transport machinery to ensure delivery of the incoming viral genomes to the trans-Golgi network^35^. Upon entry into mitosis, the nuclear envelope is lost and the viral L2/DNA complex gains access to the nucleus where it resides in PODs and where initiation of viral gene expression then takes place^7,8^. The success of this entry process is thus intimately linked to the onset of mitosis^24,25^. Some major questions arise from those studies. In particular, how long can the incoming HPVs remain viable within the infected cell prior to the onset of mitosis and where do they reside during this period? Here we have attempted to provide some insights into these questions. Using a well-established model of reversible cell senescence^31^, we have been able to show that incoming HPV-16 PsVs can remain viable for up to 13 days post-exposure to the target cells, and can efficiently transduce cells after such a period of time if the cells are stimulated to re-enter the cell cycle. To our surprise however, we find that these viruses are still sensitive to neutralisation by an anti-L1 neutralising antibody, indicating that the virus is located on or near the cell surface during the whole of this period and is not retained in an intracellular vesicular compartment. These results obviously have major implications for our understanding of how long viruses can remain viable without the target cell entering mitosis, but nonetheless still being accessible to antibody neutralisation.

Using the BJ model of replicative senescence we could first confirm previous studies showing that these cells gradually cease to proliferate following extended periods in tissue culture. However, cell cycle re-entry can be efficiently induced in these senescent cells following transfection of a p53 siRNA. Using this model we first compared the ability of HPV-16 PsVs to transduce a luciferase reporter construct into early passage (proliferating) and late passage (senescent) BJ cells. Not surprisingly, we found very good levels of transduction in the early passage cells, confirming that many different cell types, including fibroblasts, are susceptible to infection with HPV PsVs^36,37^. In addition, we also confirmed earlier studies demonstrating an absolute requirement for cell proliferation for the infectious entry to be successful, with only very low levels of reporter gene transduction being obtained in the senescent cell population. However, the efficacy of the model was demonstrated following transfection of the senescent cells with a p53 siRNA; this induced cell cycle re-entry and rendered the cells susceptible to infection with the HPV-16 PsVs.

Using this model of inducible cell cycle re-entry, we first investigated how long HPV-16 PsVs could remain infectious after being exposed to the cells, but prior to the cells undergoing mitosis. We found that virions were remarkably stable in these assays, and could readily attain complete infectious entry, as determined by luciferase transduction, for up to 16 days post-infection. However, by 30 days, no sign of infectivity remained, indicating that virions can remain infectious on a target cell for 2 weeks before the cell enters the cell division cycle and completes mitosis. It should be emphasised that the ability of the HPV-16 PsVs to infect these cells is not due to the residual presence of free virus in the medium, as we were never able to obtain infectious virus from the tissue culture supernatants; rather it indicates the presence of virus either strongly bound or internalised in the target cells (data not shown). At this stage we do not know the fate of the virus between the 16- and 30-day time points, as we have been unable to recover infectious virus from these cells. Future studies will be required to investigate this aspect further.

Obviously, a major question arising from this analysis was; where within the cell does the virus reside whilst the cell is in a senescent state for these extended periods of time? We provide strong evidence that under these circumstances, HPV-16 PsVs do not actually enter an internal vesicular compartment, but remain attached to the cell surface. Several lines of evidence support this conclusion. Firstly in all assays, we found that anti-L1 neutralising antibody could very effectively abolish virus infection, even in cultures where the cells had been exposed to virions nearly two weeks prior to neutralisation. Secondly, immunofluorescence analyses failed to show any co-localisation of HPV-16 PsVs with either endosomes or lysosomes in senescent cells, and such co-localisation was only observed following transfection of the cells with p53 siRNA to promote cell cycle re-entry. Indeed, in the senescent cells there was consistent indication of AF488-labelled HPV-16 PsVs accumulating on the cell surface. In agreement with this, we found that when non-fixed non-permeabilised cells were exposed to the anti-L1 neutralising antibody, this could co-localise with cell-surface-bound HPV-16 PsVs in the senescent cells. However when the same assay was performed on cells that had been stimulated to enter the cell cycle, following transfection of the p53 siRNA, there was strongly reduced co-localisation of the AF488-labelled PsVs and the anti-L1 neutralising antibody.

An important point arising from these studies was whether the senescent cells were simply defective in endocytic uptake. However in an extensive series of assays looking at transferrin uptake, we observed no major differences between the senescent cells and those that were stimulated back into cell cycle following transfection of p53 siRNA. Obviously, these studies have the caveat of being performed in fibroblasts and not in basal keratinocytes, the natural target cell for these viruses. Indeed, when comparing infectivity of early passage BJ fibroblasts with HeLa cells we do indeed see significantly lower levels of infectivity in the fibroblasts. Unfortunately we have not been able to identify conditions whereby keratinocytes can be maintained in a growth-arrested state for several weeks, making it impossible to perform similar studies in that cell setting. However, these studies nonetheless demonstrate an intriguing capacity of the virus to remain viable on the surface of senescent cells for extended periods of time, and it is reasonable to speculate that this is also relevant for HPV PsV interactions with other cell types.

Taken together, these results have a number of important implications. They once again highlight the importance of mitosis for completion of virus infection, but they also make a further compelling case that active growth-promoting signalling is required for virus uptake to actually take place. Previous studies have indicated that EGFR signalling was most likely required for virion uptake, and certainly blocking EGFR with an inhibitory antibody blocks virus entry, suggesting its possible use as an entry receptor^28,29^. Our studies do not exclude this possibility, but they strongly suggest that the proliferative signals associated with the onset of cell division are linked to the form of endocytic transport required by HPV for a successful virus infection. Which signalling pathways, or types of endocytic transport, are so linked to the cell cycle and virus entry will be a subject of future study. Finally these studies also highlight that HPV-16 PsVs are remarkably stable when bound to their target cell, and can remain infectious for several weeks. Nonetheless during the whole of this time the virus is highly vulnerable to neutralising antibodies. This suggests that, from a virological perspective, residing in an internal vesicular compartment for extended periods of time is not compatible with virus survival, but rather, being bound to the cell surface and primed to enter the cell upon the receipt of appropriate growth-promoting signals is most favourable.

## Electronic supplementary material

Supplementary informations
