# Supplementary material for: HPV-16 virions can remain infectious for 2 weeks on senescent cells but require cell cycle re-activation to allow virus entry
Source: Sci Rep. 2018 Jan 16;8:811. doi: 10.1038/s41598-017-18809-6 (PMC5770383; doi:10.1038/s41598-017-18809-6)
Supplement: Supplementary file 1 — Supplementary informations [file 41598_2017_18809_MOESM1_ESM.pdf]

HPV-16 virions can remain infectious for 2 weeks on senescent cells but require cell cycle re-activation to allow virus entry.

Justyna Broniarczyk[1,2] ^, Nadja Ring[1] ^, Paola Massimi[1], Mauro Giacca[1] and Lawrence Banks[1]*

[1]Tumour Virology Laboratory, International Centre for Genetic Engineering and Biotechnology, Padriciano 99, I-34149 Trieste, Italy, [2]Department of Molecular Virology, Adam Mickiewicz University, Umultowska 89, 61-614 Poznan, Poland

Key Words: Human Papillomavirus, Infectious Entry, Senescence

^Equal First Authors.

* Author for Correspondence.

Lawrence Banks
International Centre for Genetic Engineering and Biotechnology,
Padriciano 99,
I-34149 Trieste, Italy
banks@icgeb.org

SENESCENT STORY

2/11/17

sip53 / on WB in BJ sensencent cells

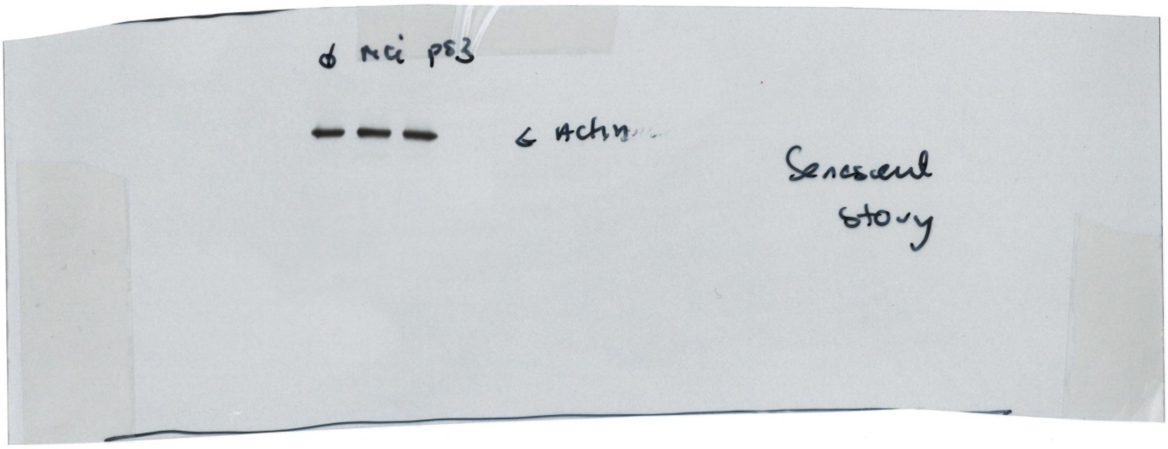

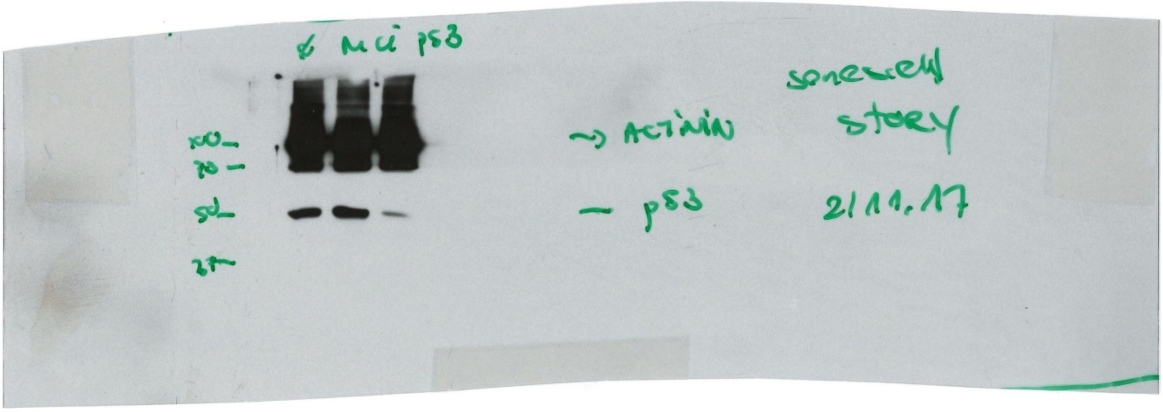

- 6 well plates
- 100uL RIPA for lysis

→ 60uL + 10uL 6x LB on gel

WB Actin 1:1000 Rb
p53(KO)mouse 1:1000
